# Bright ferritin for long-term MR imaging of human embryonic stem cells

**DOI:** 10.1186/s13287-023-03565-4

**Published:** 2023-11-14

**Authors:** Keyu Zhuang, Rocco Romagnuolo, Tamilla Sadikov Valdman, Kyle D. W. Vollett, Daniel A. Szulc, Hai-Ying Mary Cheng, Michael A. Laflamme, Hai-Ling Margaret Cheng

**Affiliations:** 1 Institute of Biomedical Engineering, University of Toronto, Toronto, ON Canada; 2 Translational Biology and Engineering Program, Ted Rogers Centre for Heart Research, 661 University Avenue, Room 1433, Toronto, ON M5G 1M1 Canada; 3 McEwen Stem Cell Institute, University Health Network, Toronto, ON Canada; 4 Department of Biology, University of Toronto Mississauga, Mississauga, ON Canada; 5 Department of Cell and Systems Biology, University of Toronto, Toronto, ON Canada; 6 Peter Munk Cardiac Centre, University Health Network, Toronto, ON Canada; 7 Department of Laboratory of Medicine and Pathobiology, University of Toronto, Toronto, ON Canada; 8 The Edward S. Rogers Sr. Department of Electrical and Computer Engineering, University of Toronto, Toronto, ON Canada

**Keywords:** Molecular imaging, Cell tracking, Reporter genes, Ferritin, Human embryonic stem cell

## Abstract

**Background:**

A non-invasive imaging technology that can monitor cell viability, retention, distribution, and interaction with host tissue after transplantation is needed for optimizing and translating stem cell-based therapies. Current cell imaging approaches are limited in sensitivity or specificity, or both, for in vivo cell tracking. The objective of this study was to apply a novel ferritin-based magnetic resonance imaging (MRI) platform to longitudinal tracking of human embryonic stem cells (hESCs) in vivo.

**Methods:**

Human embryonic stem cells (hESCs) were genetically modified to stably overexpress ferritin using the CRISPR-Cas9 system. Cellular toxicity associated with ferritin overexpression and manganese (Mn) supplementation were assessed based on cell viability, proliferation, and metabolic activity. Ferritin-overexpressing hESCs were characterized based on stem cell pluripotency and cardiac-lineage differentiation capability. Cells were supplemented with Mn and imaged in vitro as cell pellets on a preclinical 3 T MR scanner. T1-weighted images and T1 relaxation times were analyzed to assess contrast. For in vivo study, three million cells were injected into the leg muscle of non-obese diabetic severe combined immunodeficiency (NOD SCID) mice. Mn was administrated subcutaneously. T1-weighted sequences and T1 mapping were used to image the animals for longitudinal in vivo cell tracking. Cell survival, proliferation, and teratoma formation were non-invasively monitored by MRI. Histological analysis was used to validate MRI results.

**Results:**

Ferritin-overexpressing hESCs labeled with 0.1 mM MnCl_2_ provided significant T1-induced bright contrast on in vitro MRI, with no adverse effect on cell viability, proliferation, pluripotency, and differentiation into cardiomyocytes. Transplanted hESCs displayed significant bright contrast on MRI 24 h after Mn administration, with contrast persisting for 5 days. Bright contrast was recalled at 4–6 weeks with early teratoma outgrowth.

**Conclusions:**

The bright-ferritin platform provides the first demonstration of longitudinal cell tracking with signal recall, opening a window on the massive cell death that hESCs undergo in the weeks following transplantation before the surviving cell fraction proliferates to form teratomas.

**Supplementary Information:**

The online version contains supplementary material available at 10.1186/s13287-023-03565-4.

## Background

Stem cell therapy is an emerging therapeutic paradigm, with the putative ability to regenerate all cell types in the body. For the heart, brain, and spinal cord, the notion of using stem cells to replace injured or diseased tissue is uniquely relevant, since these organs have very low regenerative capacity in the adult human [1]. Yet, in practice, low cell survival rates, limited cell growth or maturation, and tenuous relationships with improved tissue function present significant obstacles to therapeutic success. A major current limitation is our inability to probe the fate of stem cells once they are transplanted in vivo. Histology for validating the surviving cell fraction and tissue growth can be employed in animals, but this approach fails to provide time-course information. If working with small animals, in vivo bioluminescence imaging (BLI) with luciferase may be utilized for cell tracking [2], but, as with histology, this method has no translational relevance. To truly enable cell therapy optimization and translation, a non-invasive cell tracking technology is needed to inform on cell survival, distribution, and proliferation in deep tissue.

In vivo cell tracking in humans has been reported using magnetic resonance imaging (MRI) with superparamagnetic iron oxide (SPIO)-labeled cells and positron emission tomography and single photon emission computed tomography (PET/SPECT) imaging with radiotracer-labeled cells [3–5]. However, the exogenous cell labeling employed is far from ideal: label retention is short-term (days), label dilutes as cells divide, and label can be non-specific due to uptake by infiltrating macrophages [1, 6, 7]. With PET/SPECT, there is the additional drawback of radiation dose and very short label half-lives. To achieve long-term cell tracking, a reporter gene strategy that incorporates the transgene of interest into the genome of the cell population is more effective. Ferritin, an ubiquitous iron storage protein critical to iron homeostasis, has been described as a reporter gene for in vivo cell tracking on MRI [8]. Cells made to overexpress ferritin can generate dark contrast via intracellular iron accumulation [8, 9]. However, its very low sensitivity remains a major challenge, as the iron-induced contrast generated by ferritin-overexpressing cells is generally very small, with contrast efficiency varying across different cell types and disease models [8–11]. There is also the general ambiguity of dark contrast and presence of blooming artifacts, which are exacerbated in damaged tissue where hemosiderin deposits can also create hypointense signal [10].

Our group has previously reported a novel bright ferritin-based MRI cell tracking platform that overcomes the limited sensitivity and specificity of the conventional ferritin mechanism [12]. It was dubbed “bright ferritin” because of the discovery that manganese (Mn) generated intracellular Mn-ferritin nanoparticles that imparted bright contrast. In contrast to conventional ferritin, which yielded no significant dark contrast change in transplanted human embryonic kidney (HEK) cells, the bright-ferritin platform provided specific and sustained T1-weighted positive contrast readily distinguished from surrounding tissue. In this study, we explore the bright ferritin platform in human embryonic stem cells (hESCs), a main cell source in regenerative medicine owing to its capability of unlimited self-renewal and differentiation into all cell types in the body [13]. The objective of this study was two-fold: (1) to determine if ferritin-overexpressing hESCs retained cell function, stemness, and tumorigenicity, and (2) to investigate the feasibility of using bright ferritin to monitor hESC survival, distribution, and proliferation in vivo.

## Methods

### Animals

This study was approved by the University of Toronto’s Animal Care Committee (protocol #20012748). All procedures were conducted in accordance with the Canadian Council on Animal Care. Two-month-old female NOD SCID (n = 15) and CD-1 (n = 12) mice (Charles River Laboratories, Canada) weighing between 20 and 25 g were utilized. The reporting of animal experiments adheres to the ARRIVE guidelines (https://arriveguidelines.org/arrive-guidelines).

### Human embryonic stem cell (hESC) cell culture and maintenance

ESI-017 hESCs (ESI BIO, SKU: ES-700; Alameda, CA, USA) [13] were cultured on Matrigel™ hESC-qualified Matrix (Corning; Corning, NY, USA) coated tissue culture plates using mTeSR™ Plus medium (StemCell Technologies; Vancouver, BC, Canada) in an incubator set at 37 °C and 5% CO_2_. Cells were maintained in an undifferentiated state as colonies and passaged using ReLeSR™ (StemCell Technologies).

### Generation of ferritin-overexpressing hESCs

A non-viral CRISPR/Cas9 gene editing system was used to integrate the human ferritin transgene into the safe-harbor locus AAVS1 of ESI-017 hESCs as previously described [12]. Transfected cells were sorted for eGFP expression before expansion of single colonies to produce clonal cell lines. Clones were selected based on the ferritin expression level determined by Western blot.

### Cardiac differentiation and flow cytometry

Human embryonic stem cell-derived cardiomyocytes (hESC-CMs) were differentiated as previously described [14, 15]. In brief, hESCs were cultured as single cells in mTeSR™ One medium (StemCell Technologies) with 10 µM ROCK inhibitor Y27632 (StemCell Technologies) until ready for embryoid bodies (EBs) generation. EBs were formed by culturing cells at a density of 1 × 10^6^ cells/ml on an orbital shaker (45 rpm) overnight in a low oxygen environment (5% CO_2_, 5% O_2_, 90% N_2_) with aggregation medium. The aggregation medium contained STEMPRO 34 medium (Thermo Fisher Scientific; Waltham, MA USA), L-glutamine (2 mM, Thermo Fisher Scientific), transferrin (150 μg/mL, Roche; Basel, Switzerland), L-ascorbic acid (50 μg/mL, Sigma-Aldrich; St. Louis, MO, USA), monothioglycerol (MTG, Sigma-Aldrich), bone morphogenetic protein-4 (BMP4, 1 ng/mL, R&D Systems; Minneapolis, MN, US), and ROCK inhibitor Y-27632 (10 μM, StemCell Technologies). EBs were then treated with a growth factor based three-stage induction using BMP4 (10 ng/ml, R&D Systems), fibroblast growth factor basic (bFGF, 5 ng/ml, R&D Systems), and activin A (6 ng/ml, R&D Systems) for 2 days, then Wnt inhibitor IWP2 (2 μM, Tocris; Bristol, UK) and vascular endothelial growth factor (VEGF, 10 ng/mL, R&D Systems) for 3 days. The aggregates were dissociated into single cells on Day 6 of differentiation and plated into Growth Factor Reduced Matrigel (Corning)-coated flasks at a density of 80,000 cm^2^ in presence of VEGF (5 ng/ml) for another 6 days. Starting Day 12 of differentiation, cells were cultured in RPMI 1640 medium (Thermo Fisher Scientific) with B-27 supplement (Thermo Fisher Scientific) until Day 17 when the cells were cryopreserved. hESC-CM populations were assessed using a LSRII/Fortessa flow cytometer (BD Bioscience; San Jose, CA, USA) with primary antibodies against cardiac troponin T (cTnT) and myosin light chain 2 (MLC2v).

### Western blot

Cells were washed with phosphate buffer saline (PBS) and lysed on ice with lysis buffer (25 mM Tris–HCl pH 7.6, 150 mM NaCl, 1% Triton X-100, 0.5% sodium deoxycholate, and protease inhibitor). Lysates were collected and centrifuged at 15,000 g for 15 min at 4 °C. Supernatants were collected, and protein concentration was measured using Pierce BCA Protein Assay Kit (Thermo Fisher Scientific). For each sample, an equal amount of protein was mixed with 4 × Laemmili Sample Buffer (BioRad; Hercules, CA, USA), 10 mM dithiothreitol (DTT, Sigma-Aldrich), and boiled at 95 °C for 5 min. Proteins were resolved by sodium dodecyl-sulfate polyacrylamide gel electrophoresis (SDS-PAGE) and then transferred onto polyvinylidene difluoride (PVDF) membranes. Membranes were blocked with 3% bovine serum albumin (BSA) in Tris-buffered saline (TBS) + 0.05% Tween-20 for 1 h at room temperature, incubated overnight at 4 °C with primary antibodies (anti-ferritin and anti-β-actin antibodies, Abcam; Cambridge, United Kingdom), followed by horseradish peroxidase (HRP)-conjugated secondary antibodies (Abcam) for 1 h at room temperature. The chemiluminescent signals were developed by Clarity Western ECL Substrate (Bio-Rad) and imaged on a ChemiDoc™ imaging system (Bio-Rad).

### Cellular toxicity assays

To investigate cellular toxicity associated with ferritin overexpression and Mn supplementation, we assessed cells for viability, proliferation, and metabolic activity. Wild type and ferritin-overexpressing hESCs were seeded on 24-well and 96-well plates at a density of 40,000 cells/cm^2^. Twenty-four hours after seeding, cells were dosed with MnCl_2_ for 24 h. After dosing, cells were washed twice with normal culture medium. LIVE/DEAD™ Viability/Cytotoxicity Kit (Invitrogen; Waltham, MA, USA) was used to qualitatively assessed cell viability. Calcium AM, ethidium homodimer-1, and Hoechst 33342 (Invitrogen) were added to the cells to label live cells, dead cells, and cell nuclei, respectively. Representative images were captured using a Leica DMi8 inverted epifluorescence microscope. Cell proliferation and metabolic activity were assayed using CyQUANT™ NF Cell Proliferation Assay (Invitrogen) and WST-1 reagent (ROCHE; Basel, Switzerland), respectively. The reagents were added to cells according to the manufacturer instructions. The absorbance or the fluorescence intensity was measured using a PerkinElmer Envision 2104 Multilabel Plate Reader.

### Immunofluorescence microscopy

To evaluate pluripotency markers in hESCs and cardiac markers in hESC-CMs, cells were cultured on Matrigel-coated glass coverslips in 24-well plates for immunofluorescence imaging. Cells were fixed in 4% paraformaldehyde for 15 min, permeated in 0.1% Triton for 5 min, blocked with 3% BSA at 37 °C for 20 min, and incubated with primary antibodies (anti-OCT4, anti-SSEA4, anti-cTnT, anti-sarcomeric-α-actinin antibodies, Abcam; Cambridge, United Kingdom) at room temperature for 1 h. Cells were then blocked with 10% donkey serum for 30 min and incubated with Alexa Fluor conjugated secondary antibodies (Thermo Fisher Scientific) at room temperature for 1 h. Cell nuclei were stained with Hoechst 33342 (Invitrogen) for 5 min. The coverslips were mounted and imaged using an Olympus FLUOVIEW FV3000 laser scanning confocal microscope.

### Inductively coupled plasma atomic emission spectroscopy (ICP-AES) quantification of Mn content

Cells with or without MnCl_2_ supplementation were washed with PBS before collection. The cell pellets were digested with 35% HNO_3_ with sonication at 60 °C for 3 h. Samples were topped up with distilled deionized water (ddH_2_O) to a final concentration of 2% HNO_3_ and filtered through 0.22 µm membranes. Samples were stored at 4 °C before analysis on an Optima 7300 DV ICP-AES spectrometer at the Department of Chemistry, University of Toronto (Toronto, Canada).

### In vitro MRI

Cells with or without MnCl_2_ supplementation were washed with PBS before collection. Cells were then dissociated, centrifuged at 300 g for 5 min, washed with PBS, and transferred into wells in a pre-cut 96-well PCR microplate. The microplate was centrifuged at 300 g for 5 min and the supernatant was removed. Cell pellets were kept on ice until MRI on a preclinical 3 T MR scanner (MR Solutions; Guildford, United Kingdom).

MRI was performed by placing the microplate in the center of a mouse whole-body coil. A 2D T1-weighted spin echo (SE) sequence was run with the following parameters: repetition time (TR) = 500 ms, echo time (TE) = 11 ms, matrix = 256 × 256, field-of-view (FOV) = 50 × 50 mm, slice thickness = 1.0 mm, voxel size = 0.1953 × 0.1953 × 1.0 mm^3^, number of signal averages (NSA) = 4. T1 mapping was performed using a variable flip angle approach [16] with a 3D spoiled gradient echo (SPGR) sequence: TR = 11 ms, TE = min, flip angle (FA) = [2°, 3°, 10°, 20°], matrix = 256 × 128 × 12, FOV = 50 × 50 mm, slice thickness = 1.0 mm, voxel size = 0.195 × 0.390 × 1.0 mm^3^, NSA = 4.

### In vivo MRI

On the day of cell transplantation, hESCs were dissociated from 10 cm dishes using ReLeSR, washed with PBS, centrifuged at 300 g for 5 min, and kept on ice until ready for cell injection. Cells were manually counted with a hemocytometer. For each leg in each animal, 3 × 10^6^ cells in a volume of 50 µl were injected into the gastrocnemius muscle using a 1 ml syringe and 27 G needle. After the baseline MRI scan, 0.4 mmol/kg of Mn was administrated subcutaneously (for a mouse weighing 25 g, 100 µl of 0.1 M MnCl_2_ solution was injected).

Mice were induced on 5% isoflurane in 100% oxygen (1.0 L/min), maintained at 1.5–2.0% isoflurane during the entire imaging session, and placed head-first in a prone position inside the mouse whole-body coil. Body temperature was maintained at 37 °C using an air-heating system built into the mouse holder. A pneumatic respiratory pillow was placed on the abdomen to monitor respiration throughout imaging and to provide respiratory gating. A high-resolution 2D fat-suppressed T1-weighted fast spin echo (FSE) sequence was run with the following parameters: TR = 743 ms, TE = 11 ms, echo train length (ETL) = 4, matrix = 256 × 248, FOV = 60 × 45 mm, slice thickness = 1.0 mm, voxel size = 0.234 × 0.181 × 1.0 mm^3^, and NSA = 2. T1 mapping was performed using a variable flip angle approach [16] with a 3D SGPR sequence: TR = 11 ms, TE = “MIN”, FA = [2°, 10°, 20°], matrix = 256 × 128 × 24, FOV = 60 × 45 mm, slice thickness = 1.0 mm, voxel size = 0.234 × 0.351 × 1.0 mm^3^, and NSA = 4. A high-resolution 2D T2-weighted FSE sequence was also run to exclude fluid contributions either from the injectate or from inflammation: TR = 3658 ms, TE = 68 ms, ETL = 8, matrix = 256 × 240, FOV = 60 × 45 mm, slice thickness = 1.0 mm, voxel size = 0.234 × 0.187 × 1.0 mm^3^, and NSA = 2. MRI was performed daily during the first five days post-cell transplantation and thereafter every other week (Day 1–5, Week 2, Week 4, and Week 6, etc.). After the last imaging session, NOD SCID mice were euthanized by cardiac arrest with 10% potassium chloride injection into the right ventricle under anesthesia with 5% isoflurane in 100% oxygen. CD-1 mice were euthanized by cardiac puncture under anesthesia with 5% isoflurane in 100% oxygen.

### MRI data analysis

Quantitative T_1_ data analysis was performed in-house using Matlab (v2022, MathWorks, Natick, MA, USA). Regions of interest (ROIs) were drawn manually around the enhancing region on T1-weighted FSE. T_1_ relaxation times were averaged across the ROI to arrive at a mean value.

### Histological analysis

The leg muscles were fixed with 4% paraformaldehyde, processed, and paraffin-embedded for histological analysis. Sections 5 µm thick were stained using hematoxylin and eosin (H&E, Newcomer Supply; Middleton, WI, USA), immunostained using anti-Ku80 antibody (Cell Signalling Technology; Danvers, MA, USA), followed by HRP-conjugated secondary antibody (Abcam), developed with SignalStain DAB Substrate Kit (Cell Signalling), and counterstained with hematoxylin.

### Statistical analysis

Data are presented as mean ± SEM, except for in vitro T1 values, which are presented as mean ± SD. For in vitro studies, two-sample comparisons were made using a Welch's *t*-test. For in vivo studies, data were analyzed in Matlab using a three-way analysis of variance (ANOVA), where ferritin/wild type, teratoma formation, and time post-cell transplantation were independent variables. Fisher’s post-hoc analysis was performed for multiple comparisons. In all cases, significance was reported at a *p*-value of 5%.

## Results

**Fig. 1:**
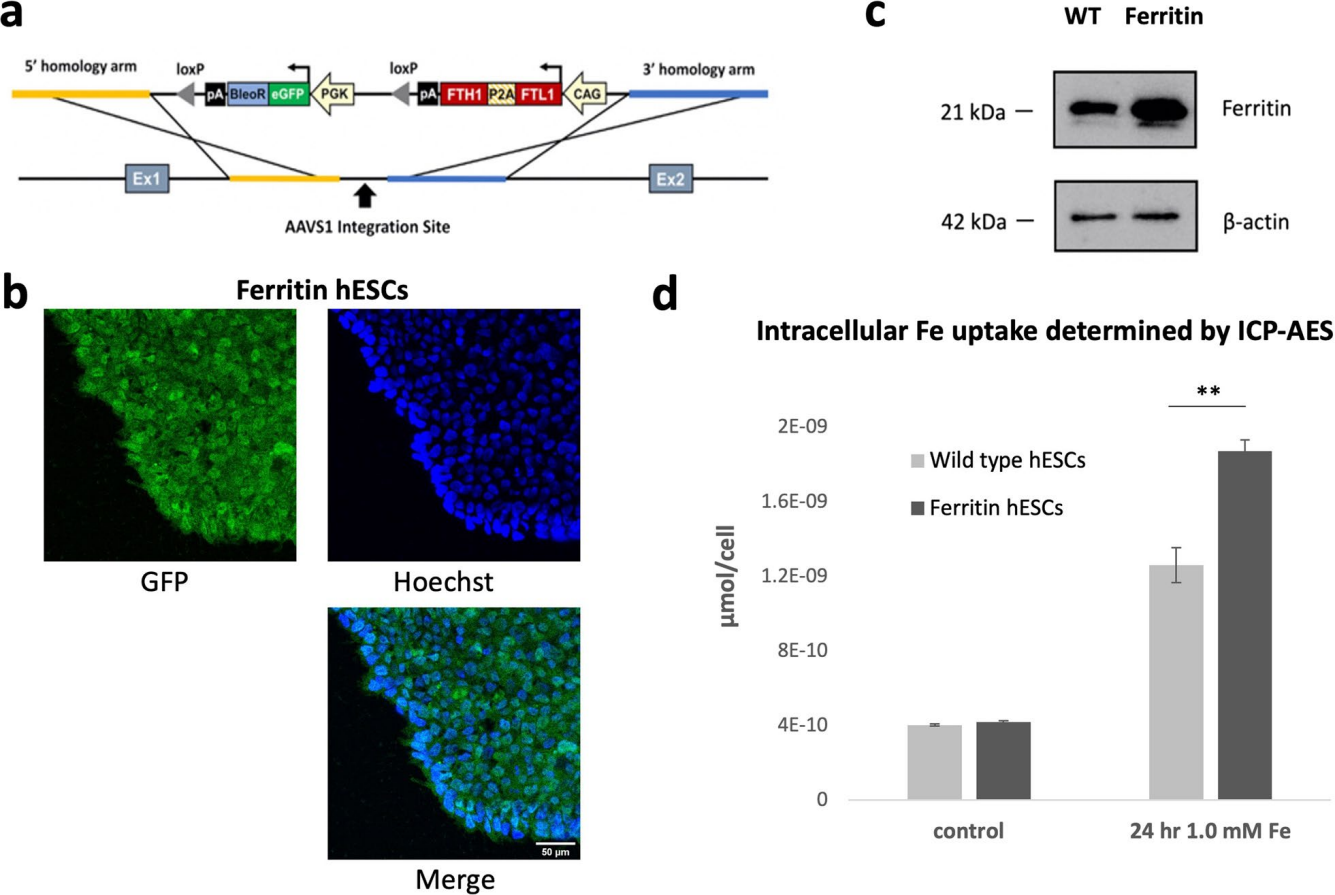
CRISPR/Cas9-engineered hESCs demonstrate stable ferritin overexpression. **a** Schematic of the plasmid vector for inserting the human ferritin transgene into the AAVS1 locus in the genome of hESCs ESI-017 using a non-viral CRISPR/Cas9 system. **b** Immunofluorescence imaging confirmed gene insertion with eGFP tag (green). Cell nuclei were stained by Hoechst 33342 (blue). Scale bar = 50 μm. **c** Western blot confirmed ferritin overexpression in hESCs relative to wild type (WT); β-actin was used as loading control. Full-length blots are presented in Additional file 1: Fig. S1. **d** Cellular iron uptake measured by inductively coupled plasma atomic emission spectroscopy (ICP-AES) confirmed the functionality of ferritin. Cells were supplemented with 1.0 mM ferric ammonium citrate for 24 h. Data are presented as mean ± SEM (n = 3). ** *P* < 0.01

A mutant hESC cell line was generated that stably overexpresses human ferritin (Fig. 1). As previously described [12], a single, targeted transgene was inserted at the AAVS1 safe harbour locus using the CRISPR-Cas9 system (Fig. 1a). Gene integration was confirmed by junctional PCR (data not shown) and the presence of eGFP in mutant cells (Fig. 1b). Overexpression of the ferritin protein was confirmed by Western blot, which showed a higher ferritin expression level in mutant cells compared to wild type cells (Fig. 1c). The in vitro functionality of ferritin was evaluated based on cell uptake of iron; intracellular iron content measured by ICP-AES showed a significantly higher iron content in mutant cells relative to wild type cells (*P* < 0.01) (Fig. 1d).

**Fig. 2:**
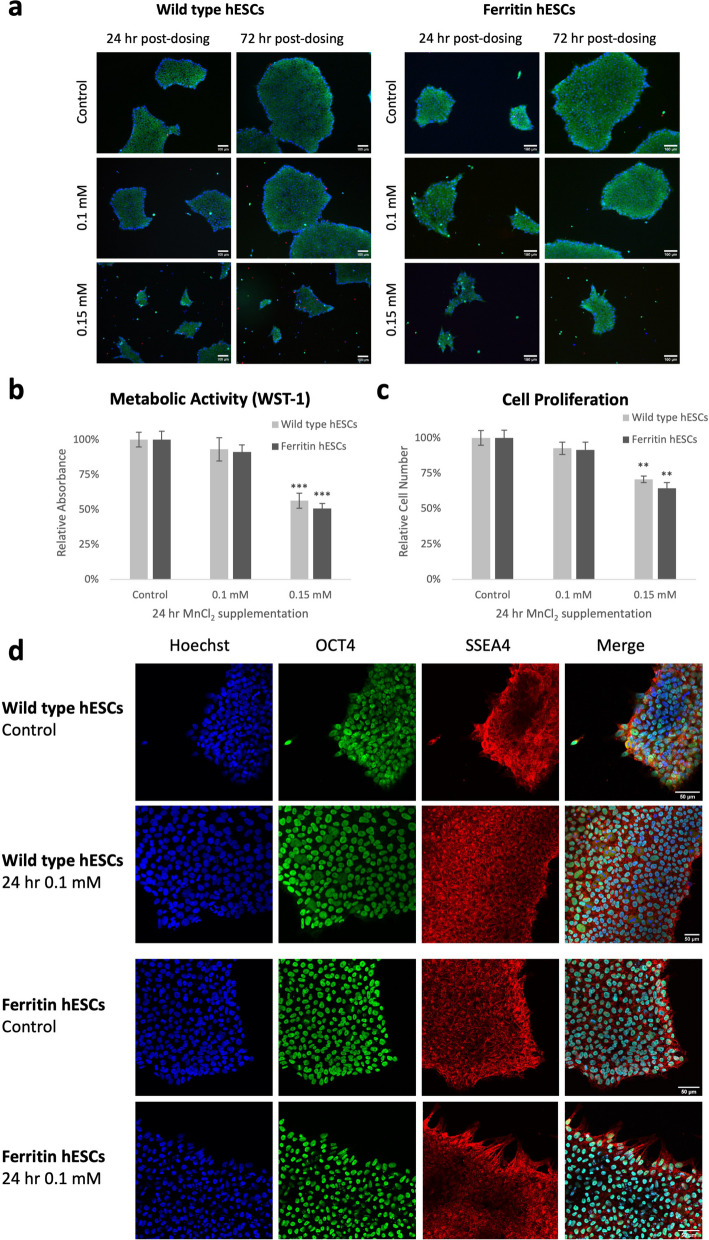
Impact of ferritin overexpression and Mn supplementation on cell viability, proliferation, and pluripotency. Cellular toxicity assessment of hESCs revealed minimal impact on cell growth, proliferation, metabolic activity, and pluripotency from ferritin overexpression and supplementation with 0.1 mM MnCl_2_ for 24 h. Significant cell death and negative impact on cell growth were noted for 0.15 mM MnCl_2_. **a** Live (green)/Dead (red) fluorescent assay performed at 24- and 72-h post-Mn supplementation for wild type and ferritin-overexpressing hESCs. Cell nuclei were stained by Hoechst 33342 (blue). Scale bar = 100 μm. **b** Metabolic (WST-1) activity and **c** cell proliferation measured by relative absorbance or fluorescence units at 24 h post-Mn supplementation. Positive controls were cultured in standard growth medium with no additional supplementation. Data are presented as mean ± SEM (n = 6). ** *P* < 0.01, *** *P* < 0.001. **d** Counterstaining for pluripotency markers transcription factor OCT4 (green) and cell surface marker SSEA4 (red) revealed minimal impact on cell pluripotency from ferritin overexpression and supplementation with 0.1 mM MnCl_2_ for 24 h. Cell nuclei were stained by Hoechst 33342 (blue). Scale bar = 50 μm

Cellular toxicity associated with ferritin overexpression or Mn supplementation was assessed next (Fig. 2). Visually, there was minimal effect on cell viability and morphology when wild type and ferritin-overexpressing mutant hESCs were dosed with 0.1 mM MnCl_2_ for 24 h. Cell death was observable only when Mn supplementation was increased to 0.15 mM (Fig. 2a). The same critical dosing point was observed in the cell proliferation and metabolic activity assays. Both wild type and ferritin-overexpressing hESCs displayed no difference as compared to positive controls when supplemented with 0.1 mM MnCl_2_ (*P* = 0.34 and 0.54, respectively), but proliferation and metabolic activity decreased when supplemented with 0.15 mM MnCl_2_ (*P* < 0.01) (Fig. 2b, c).

**Fig. 3:**
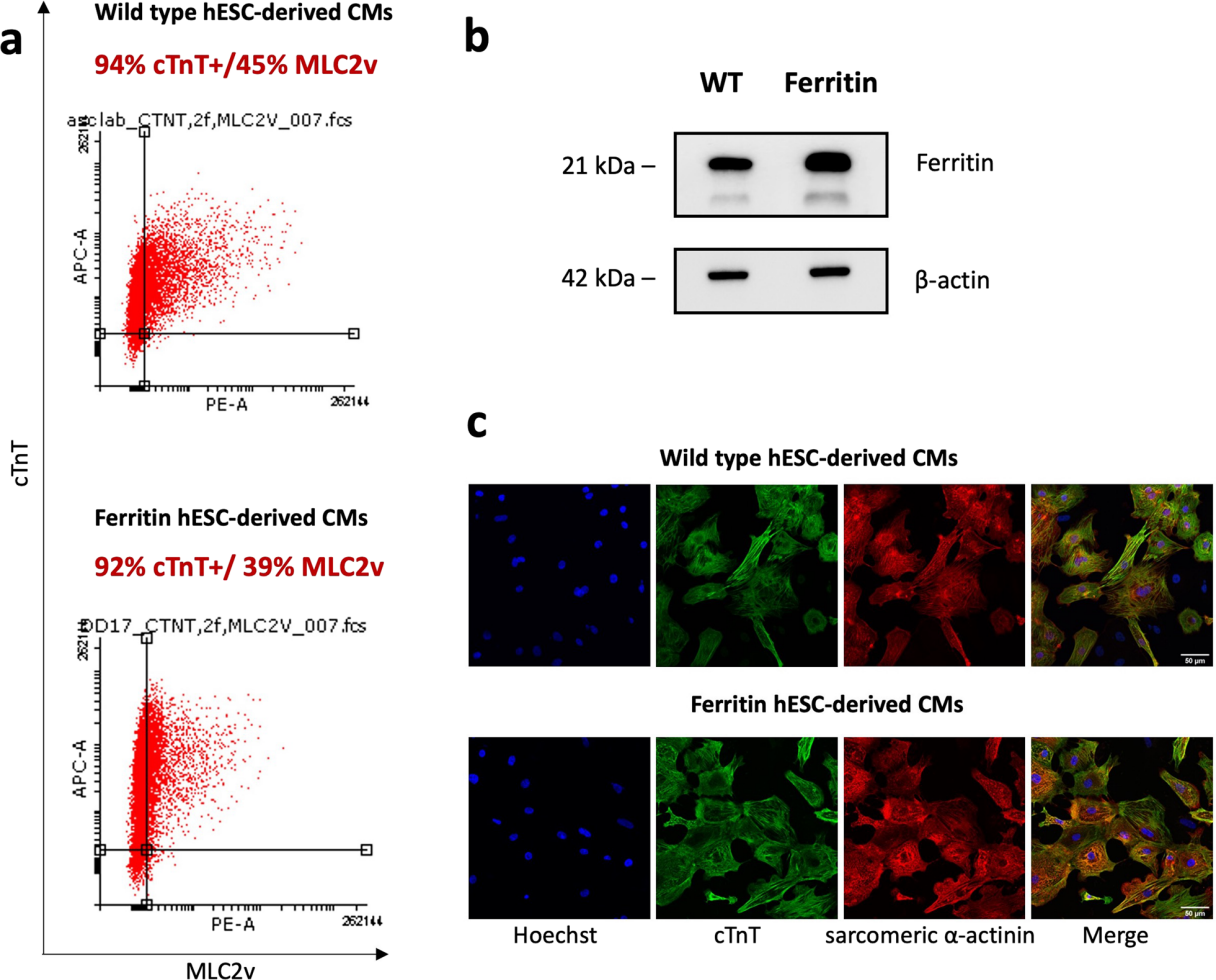
Ferritin overexpression does not affect hESC differentiation into cardiomyocytes. **a** Flow cytometry data demonstrated the purity of wild type and ferritin-overexpressing hESC-CMs. The percentage of positive cells for the cardiac marker cardiac tropnin T (cTnT) and ventricular-specific marker ventricular isoform of the myosin light chain 2 (MLC2v) was determined. **b** Western blot confirmed ferritin overexpression relative to wild type (WT) after differentiation into cardiomyocytes; β-actin was used as loading control. Full-length blots are presented in Additional file 1: Fig. S1. **c** Immunofluorescence staining of cardiac contractile proteins cTnT (green) and sarcomeric α-actinin (red) demonstrated phenotypically normal ferritin-overexpressing hESC-CMs. Cell nuclei were stained by Hoechst 33342 (blue). Scale bar = 50 μm

Having identified a safe dose limit of 0.1 mM MnCl_2_, we then assessed the pluripotency and cardiac lineage differentiation of ferritin-overexpressing hESCs. Immunostaining for the pluripotency markers OCT4 and SSEA4 confirmed that gene editing and Mn supplementation did not affect stemness (Fig. 2d). We also differentiated the hESCs into cardiomyocytes in vitro [14]. As shown in Fig. 3, ferritin overexpression was retained after cardiac lineage differentiation (Fig. 3b): ferritin-overexpressing hESC-derived cardiomyocytes displayed a high purity for the pan-cardiac marker cTnT (> 90%) by flow cytometry (Fig. 3a) and a similar morphological phenotype as wild type cardiomyocytes (Fig. 3c).

**Fig. 4:**
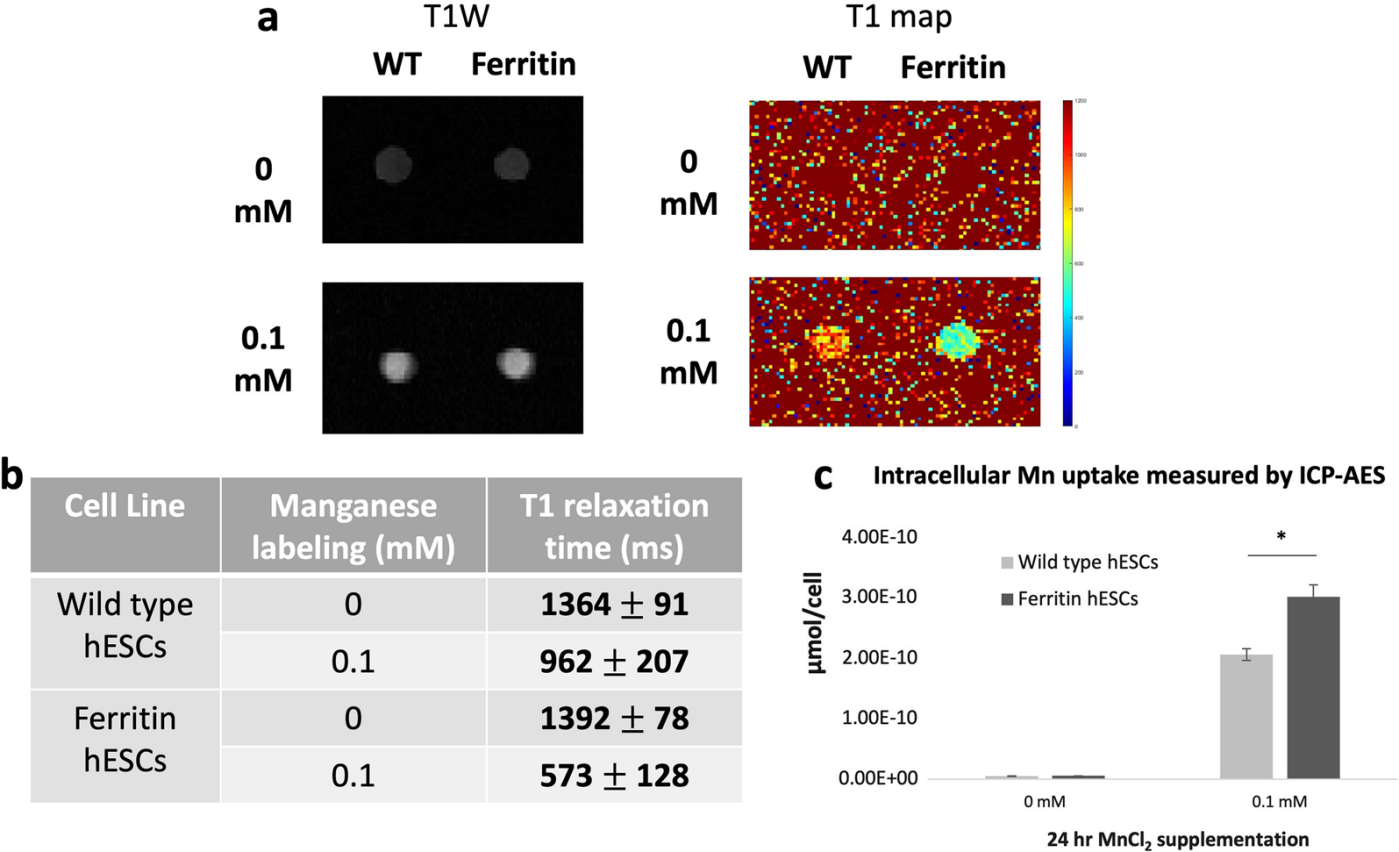
In vitro MRI demonstrates bright contrast efficiency of ferritin-overexpressing hESCs. **a** T1-weighted spin echo image (T1W) and T1 map of wild type (WT) and ferritin-overexpressing (Ferritin) hESCs supplemented with 0.1 mM MnCl_2_ for 24 h. At baseline, WT and Ferritin hESCs displayed similar contrast levels. Upon Mn supplementation, Ferritin hESCs exhibited a T1 value that was ~ 1.7-fold lower than that of WT hESCs. **b** Mean T1 values ± SD of each condition calculated from T1 maps. **c** Intracellular Mn content measured by ICP-AES, showing that Ferritin hESCs took up ~ 1.5-fold more Mn that WT cells. Data are presented as mean ± SEM (n = 3). * *P* < 0.05

In vitro MRI was performed to determine if the safe dose of 0.1 mM MnCl_2_ yielded sufficient contrast on MRI. Figure 4 illustrates cell pellets imaged using T1-weighted FSE and quantitative T1 mapping. At baseline, wild type and ferritin-overexpressing hESCs displayed similar contrast levels. Upon Mn supplementation, both cells displayed considerable bright contrast on T1w FSE image; ferritin-overexpressing cells had a 1.7-fold lower T1 relaxation time than wild type cells (Fig. 4a, b). ICP-AES quantification of Mn content revealed a 1.5-fold higher Mn content in ferritin-overexpressing hESCs compared to wild type cells (Fig. 4c).

The in vivo performance of the bright-ferritin platform was next evaluated in hESC transplants. A pilot Mn biodistribution study was first undertaken to ensure in vivo MnCl_2_ dosing was safe and provided sufficient T1-induced MRI contrast in the leg muscle (Table 1). After 0.4 mmol/kg MnCl_2_ was administered subcutaneously, the T1 relaxation time of leg muscle decreased 37.8% by 24 h, while the absolute Mn content increased from 0.002 mM (baseline) to 0.014 mM after 2 h, 0.014 mM after 6 h, and 0.027 mM after 24 h. Based on these results, we chose 24-h post-MnCl_2_ as the ideal timepoint for imaging. Note that although the contrast concentration increased nearly 14 times, the absolute Mn^2+^ concentration remained very low, much lower than the safe dose of 0.1 mM identified in vitro.

**Table 1:** Manganese (Mn) content in mouse leg muscle and Mn-induced T1 changes

Mn dose^a^ (mmol/kg)	Time post-contrast (hours)	[Mn^2+^] in leg muscle^b^ (mM)	Percentage T1 decrease
0	N/A	0.002 ± 0.0002	N/A
0.4	2	0.014 ± 0.002	20.9 ± 6.0%
	6	0.014 ± 0.003	20.8 ± 5.8%
	24	0.027 ± 0.005	37.8 ± 4.6%

^a^0.4 mmol/kg MnCl_2_ was administered subcutaneously in CD-1 mice

^b^A density of 1 g/ml was used for leg muscle. Data were presented as mean ± SEM (n = 3)

**Fig. 5:**
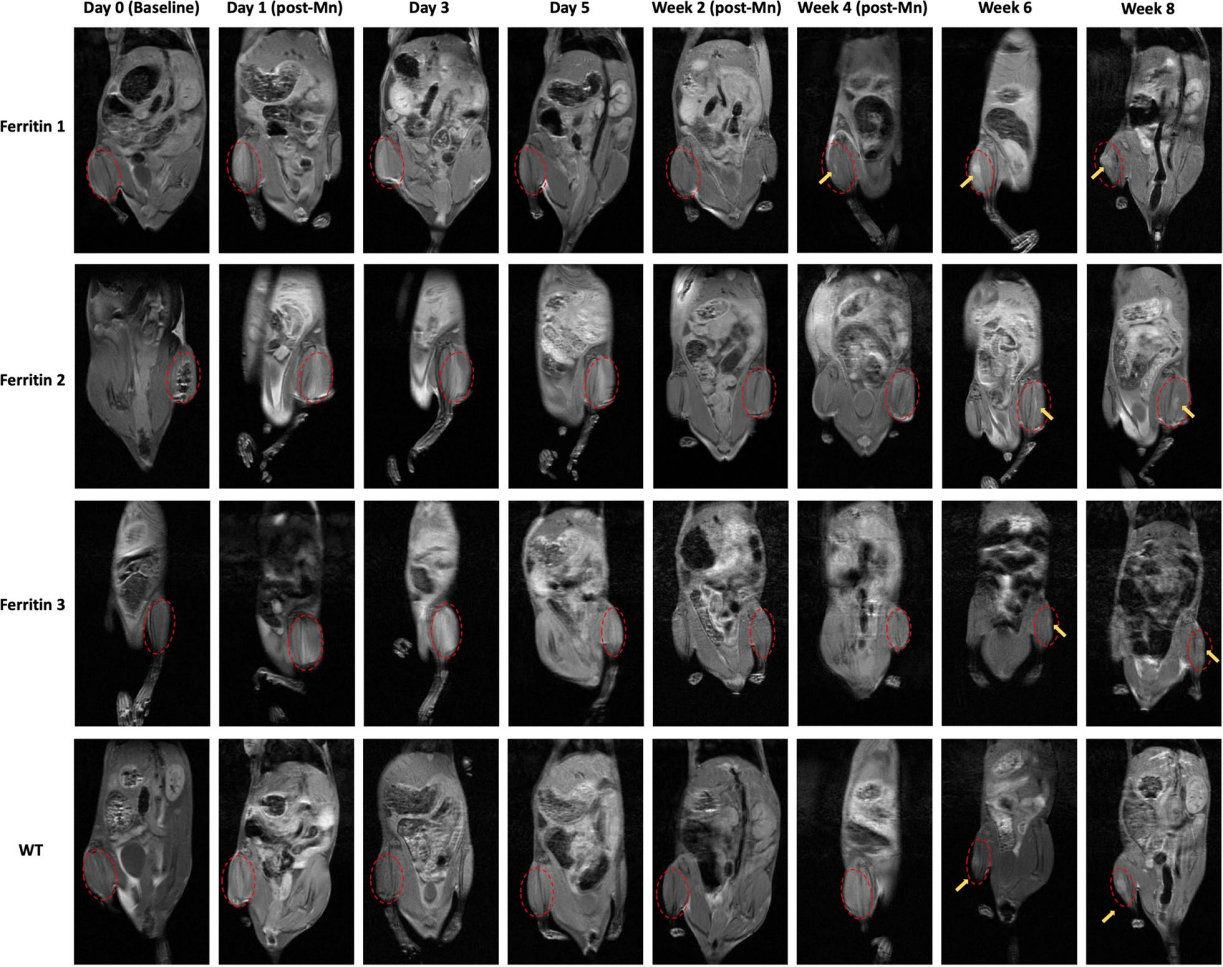
Longitudinal in vivo MRI of hESC cell injections in mice. T1w images of representative NOD SCID mice injected with 3 × 10^6^ of either ferritin-overexpressing (Ferritin) or wild type (WT) hESCs in the gastrocnemius leg muscle. Cells were injected on Day 0. Twenty-four hours after MnCl_2_ supplementation (Day 1, post-Mn), the site of cell injection (red circle) exhibited significant bright contrast, which was maintained for 5 days. Contrast had disappeared by Week 2 and recalled at Week 4 or 6 (yellow arrow) as cells began growing into a teratoma

**Fig. 6:**
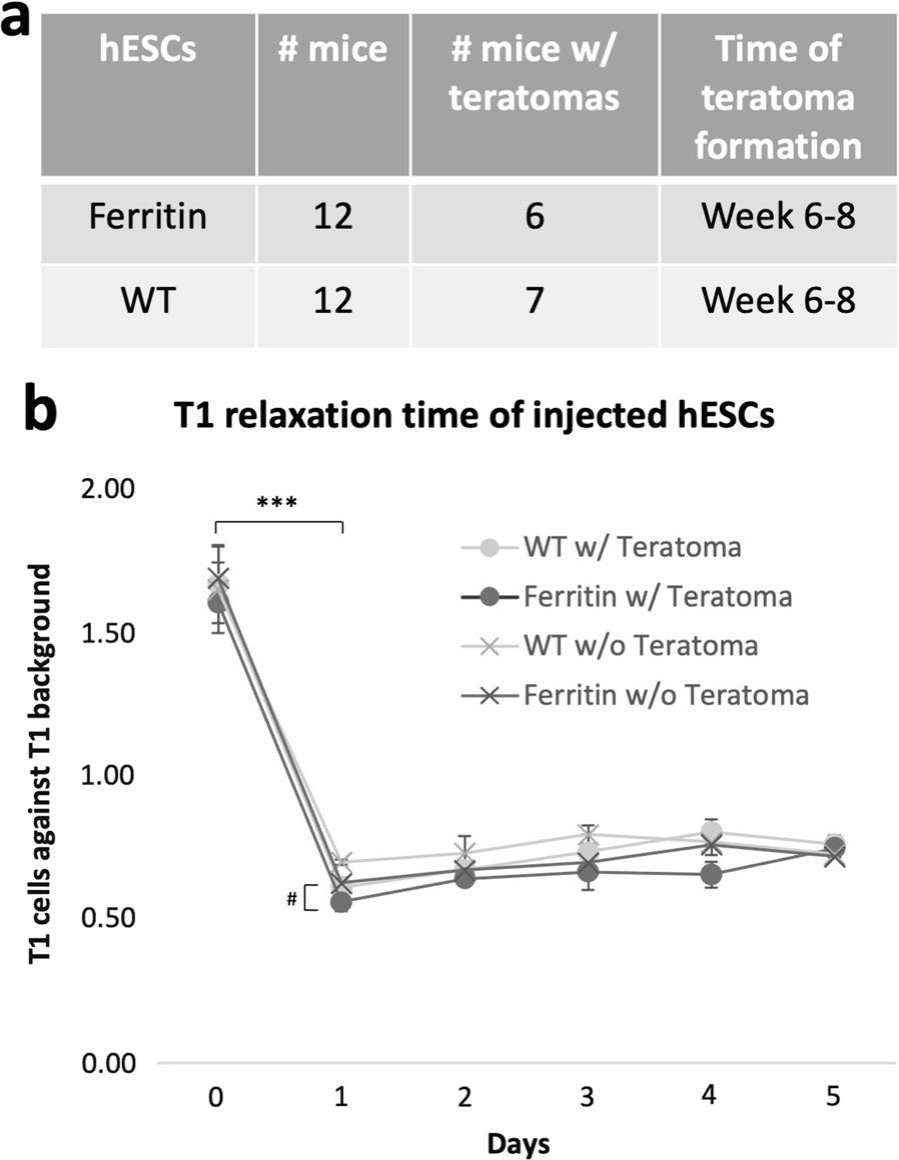
Teratoma formation efficiency and quantitative analysis for ferritin-overexpressing and wild type (WT) hESCs. **a** There was no difference in teratoma incidence between ferritin-overexpressing and wild type hESCs. **b** Quantitative T1 mapping showed that T1 in the injected cells reduced significantly after MnCl_2_ administration (Day 1) and remained low for 5 days. Data are presented as mean ± SEM. Difference in T1 between Day 0 and Day 1–5 is significant (*** *P* < 0.001). Lower T1 in ferritin cells relative to wild type where teratomas formed is noticed (# *P* = 0.086)

**Fig. 7:**
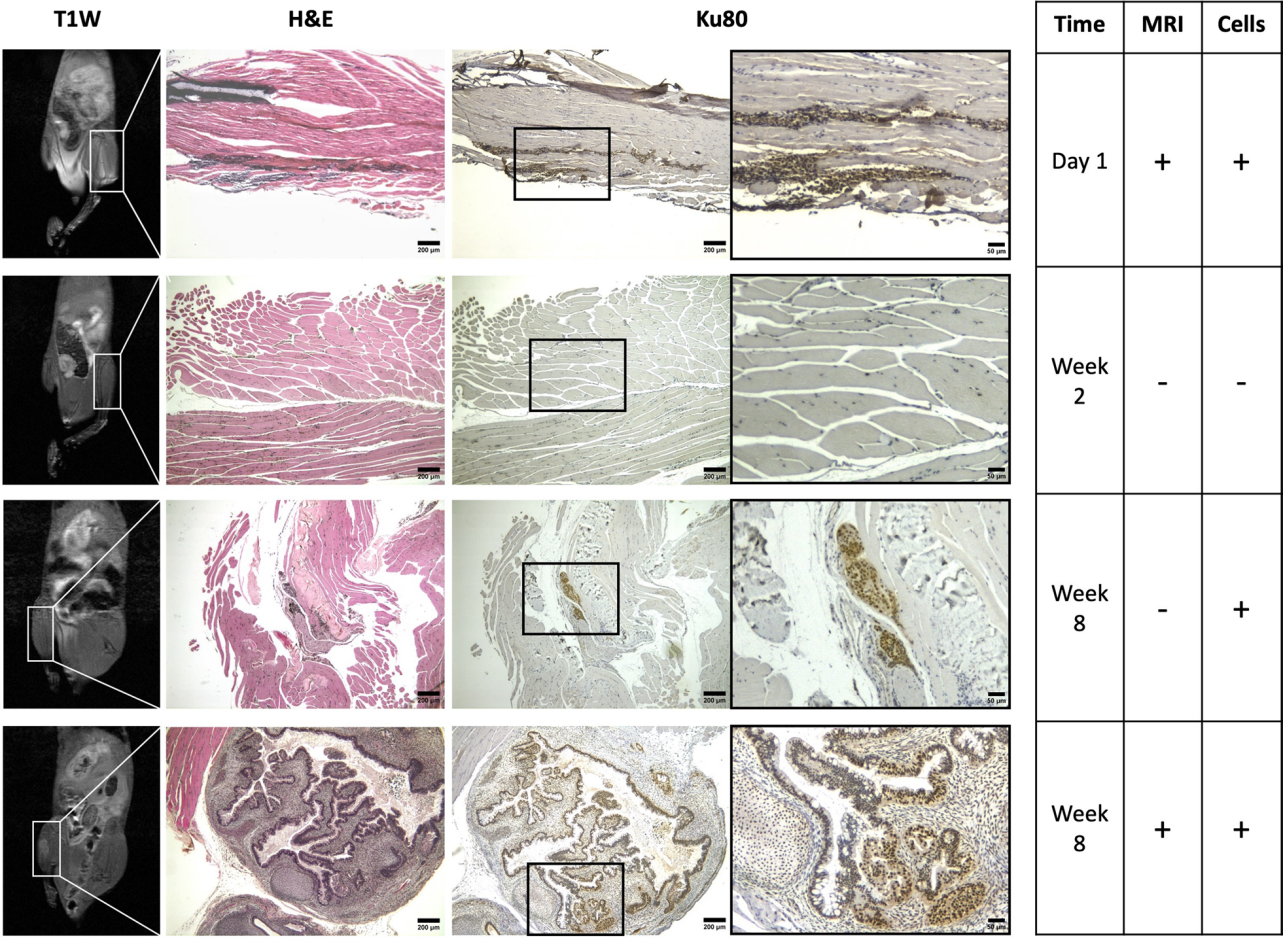
Histological analysis confirmed MRI contrast from injected cells. MRI contrast at the cell injection sites was validated by H&E staining and immunohistochemistry staining using a human-specific antibody against the nuclear antigen Ku80 (brown), counterstained with hematoxylin (purple). Four representative mice were selected to demonstrate four different observations. MRI contrast observed on Day 1 was generated by viable injected hESCs. At Week 2, no MRI contrast was observed, and no human cells were found on histology. At Week 8, MRI contrast corresponded to teratoma formation; however, in one mouse, MRI did not pick up on a very small teratoma

NOD SCID mice with ferritin-overexpressing hESCs implanted in one leg and wild type hESCs in the other were scanned repeatedly on MRI for 6 to 8 weeks to monitor cell fate (Fig. 5). At baseline (Day 0), there was no contrast at the cell injection site. Twenty-four hours after subcutaneous MnCl_2_ administration (Day 1), a diffuse bright contrast appeared in the vicinity of the cell injection site and persisted for five days. This enhancement was seen in both ferritin-overexpressing and wild type hESCs, and in keeping with in vitro results, contrast enhancement was greater in ferritin-overexpressing cells. Contrast cleared after 5 days, and bright contrast from hESCs was recalled successfully via MnCl_2_ administration beginning at 4–6 weeks after cell inoculation, depending on the animal (Fig. 5, yellow arrow). No signal was recalled in the intervening period. By 6–8 weeks, a large, fluid-filled nodule was visible, a structure later confirmed on histology to be a teratoma. There was no difference in teratoma incidence between ferritin-overexpressing and wild type hESCs (Fig. 6a). Quantitative analysis of T1 measurements revealed a substantial decrease in T1 (*P* < 0.001) after MnCl_2_ injection that was sustained for 5 days (Fig. 6b). There was also a trend towards lower T1 in ferritin-overexpressing cells relative to wild type where teratomas formed (*P* = 0.086), and lower T1 in both ferritin-overexpressing and wild type hESCs that gave rise to teratomas relative to those that did not (*P* = 0.37). Contrast observed on MRI was validated by histology on Day 1, Week 2, and Week 6–8 (depending on individual animal), confirming that bright contrast on MRI was generated by the injected human cells and emanated from a teratoma with three human germ lines (Fig. 7). Furthermore, low cell survival was confirmed on histology at Week 2, which supports the lack of bright contrast on MRI after the first week and before teratoma growth.

## Discussion

In this study, we demonstrated the ability of the bright-ferritin platform, which combines cellular overexpression of ferritin with Mn supplementation, to track hESCs sensitively and with specificity on MRI, in an on-demand and longitudinal manner. In vitro assays confirmed that ferritin overexpression did not affect cell viability, proliferation, or metabolism. Furthermore, hESCs maintained their stemness and ability to differentiate into cardiomyocytes. In vitro hESC labeling experiments revealed a 50% increase in Mn uptake by ferritin-overexpressing cells and a 70% decrease in T1 relaxation times relative to wild type hESCs. In vivo tracking of hESCs in the leg skeletal muscle of mice confirmed the presence of bright contrast in the first 5 days after cell injection. Signal was lost over the next 3–5 weeks due to low surviving cell numbers, but a small region of bright contrast was again recalled with Mn supplementation circa week 4–6 in the early days of teratoma formation, which developed 2 weeks later into a large fluid-filled structure. The ability to form teratomas was unaffected by ferritin overexpression. These in vivo results are the first demonstration in the MRI literature of on-demand signal recall using the ferritin reporter gene.

An ideal stem cell tracking platform should be biocompatible with the cell sources relevant to regenerative medicine. In the current study, we chose to demonstrate our platform on ESI-017, a clinical-grade human embryonic stem cell line manufactured under current good tissue and manufacturing practices (cGTPs and cGMPs) and fully characterized for therapeutic applications [13]. To ensure minimal impact on cell function, ferritin overexpression was achieved by making a single, targeted transgene insertion at the safe harbour locus AAVS1 to avoid potential disruption of endogenous genes [12]. This approach is much safer than viral transfection methods that enable multiple insertions at random locations in the genome [8], but it also results in a much lower expression level of the transgene. With a modest ferritin overexpression level that had no impact on ferritin function, cell viability, proliferation, stemness, and differentiation, the next step was to identify a safe Mn dose level, again based on the above properties of viability, proliferation, and stemness. At a safe in vitro dose of 0.1 mM Mn, a significant bright contrast was achieved in ferritin-overexpressing hESCs.

In vivo hESC tracking in mice confirmed the ability of the bright ferritin platform to map out the viable portion of injected cells in the days following transplantation. It also demonstrated, for the first time, the ability to recall bright contrast when a sufficient number of cells had recovered in the weeks following. Nonetheless, in vivo imaging is much more challenging than an in vitro setting. First, cells will distribute in the host tissue after transplantation, reducing cell density and further complicating sensitive detection. Second, Mn kinetics may result in very low Mn concentration in the host tissue, which was the case with skeletal muscle, where only 0.027 mM Mn^2+^ from a 0.4 mmol/kg injection had accumulated after 24 h. This implies that a very small amount of Mn was available to hESCs for uptake, which would explain why the difference in T1 between ferritin-overexpressing and wild type hESCs was much smaller in vivo than in vitro. Increasing the total dose is not an option, as that carries safety implications. However, if the hESCs had been transplanted into another tissue with higher baseline perfusion (note that skeletal muscle has one of the lowest resting perfusion of all organs), a greater T1 difference between ferritin-overexpressing and wild type hESCs would be attained.

After the first week, MnCl_2_ repeated administration could not recall bright contrast. The absence of signal is due to the low survival of hESCs, which was expected considering that up to 99% cell death is anticipated in any cell therapy [17]. Histology staining by H&E confirmed very few human cells remained at this intermediate timepoint. If we consider for a moment that 3 × 10^6^ cells were injected initially, 1% survival would leave behind 3 × 10^4^ viable cells. This number is barely larger than the limit of 10^4^ cells per voxel at the detection threshold for T1 contrast [18]. The sensitivity limit of MRI is difficult to overcome, and in any cell therapy there will be an intervening interval over which signal is lost until cell numbers recover above the threshold of detection. However, it is important to note that this shortcoming is not exclusive to MRI. Even optical imaging methods such as BLI suffer the same limitation despite its higher sensitivity: BLI cell tracking studies in mice have also shown fading of signal in the days and weeks following cell injection [2, 19].

At around 4–6 weeks post-cell injection, a small region of bright contrast could again be seen upon MnCl_2_ administration as a teratoma began to form and exponentially grow over the next couple of weeks. This teratoma assay is the ultimate test of “stemness”, and it is noteworthy that ferritin-overexpressing cells did not lose their tumorgenicity. The onset and time course of teratoma formation was also consistent with that in the literature [19–21]. Another interesting observation was a lower albeit non-significant T1 in hESCs that eventually gave rise to teratomas relative to those that did not. This lower T1 may indicate a greater surviving cell fraction in the first week, which could bias the cells accordingly towards teratoma formation.

A comparison between the present in vivo results in hESCs and those in a previous study on HEK cells [12] reveals stark differences in bright contrast that is cell-type dependent. In vivo HEK cells exhibited nearly a threefold increase in T1 relaxation rate compared to wild type, whereas hESCs exhibited a 1.1-fold increase. This discrepancy can be explained by a higher background ferritin expression in hESCs. Different cell types are known to have varying levels of ferritin expression. However, this is not a limitation for hESCs, because undifferentiated stem cells are rarely used in regenerative medicine applications. More commonly, hESCs are fully or partially differentiated towards a particular lineage and then transplanted into a subject for further maturation. For the purpose of this paper, we used hESCs to prove that ferritin overexpression had no impact on its stemness or ability to form teratomas, and to demonstrate that signal could be recalled on demand in a longitudinal manner. In future applications, the mutant hESCs would need to be differentiated for transplantation, and it is the differentiated cell for which its background ferritin expression bears relevance to the bright contrast attainable.

### Limitations

The bright ferritin platform provides a possible solution for long-term in vivo stem cell tracking, but there are several considerations that one needs to account for. The cell of interest should not in its wild type form express an inherently high level of ferritin, as that may introduce an unacceptably high background signal. Sensitivity is also limited if the surviving cell fraction drops precipitously. Therefore, this platform is best suited to cells that can survive or proliferate in vivo. Independent of cell type, the resting perfusion of the host tissue is also important, as higher perfusion facilitates delivery of supplemental Mn to the cells of interest. Based on in vitro findings, exposure to higher Mn dosing would increase the contrast separation between ferritin-overexpressing and wild type cells and, therefore, detection sensitivity.

## Conclusion

We have demonstrated the application of a previously reported bright ferritin platform for tracking hESCs longitudinally using T1-weighted contrast on MRI. In vivo and in vitro results confirm no adverse impact on cell function, stemness, differentiability, and ability to form teratomas. Longitudinal cell tracking in mice revealed a steep decline in surviving hESCs after one week and a return of bright contrast at 4–6 weeks, coincident with the early teratoma formation. This is the first demonstration of in vivo signal recall for the ferritin reporter gene.

### Supplementary Information

**Additional file 1: Fig. S1.** Corresponding full-length blots.

## Data Availability

The datasets used and/or analysed during the current study are available from the corresponding author on reasonable request.

## Abbreviations

BLI: Bioluminescence imaging
cTnT: Cardiac troponin T
EBs: Embryoid bodies
HEK: Human embryonic kidney
hESCs: Human embryonic stem cells
hESC-CMs: Human embryonic stem cell-derived cardiomyocytes
ICP-AES: Inductively coupled plasma atomic emission spectroscopy
MLC2v: Myosin light chain 2
Mn: Manganese
MRI: Magnetic resonance imaging
NOD SCID: Nonobese diabetic severe combined immunodeficiency
PET/SPECT: Positron emission tomography and single photon emission computed tomography
SPIO: Superparamagnetic iron oxide
WT: Wild type
