# Supplementary figures and images for: Bright ferritin for long-term MR imaging of human embryonic stem cells
Source: Stem Cell Res Ther. 2023 Nov 14;14:330. doi: 10.1186/s13287-023-03565-4 (PMC10647036; doi:10.1186/s13287-023-03565-4)

### Full-length Blot for Figure 1c

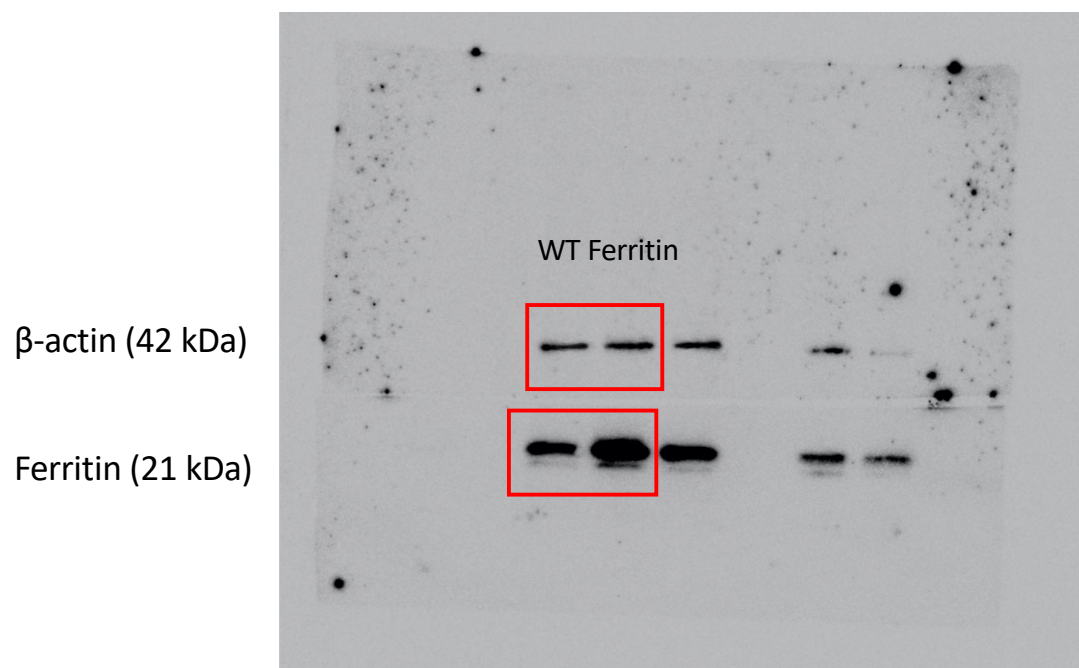

### Full-length Blot for Figure 3b

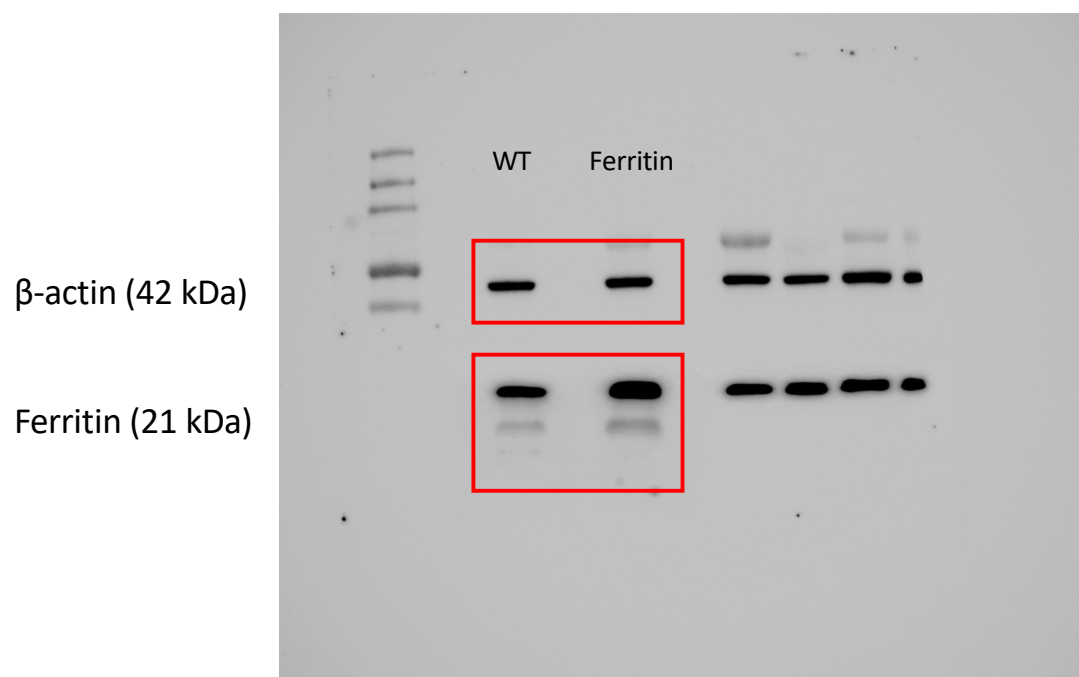

Supplement: Supplementary file 1 — Additional file 1: Fig. S1. Corresponding full-length blots. [file 13287_2023_3565_MOESM1_ESM.pdf]
